# C1D family proteins in coordinating RNA processing, chromosome condensation and DNA damage response

**DOI:** 10.1186/s13008-016-0014-5

**Published:** 2016-03-09

**Authors:** Rebecca A. Jackson, Jocelyn Shumei Wu, Ee Sin Chen

**Affiliations:** Department of Biochemistry, Yong Loo Lin School of Medicine, National University of Singapore, Singapore, 117597 Singapore; National University Health System (NUHS), Singapore, 119228 Singapore; NUS Graduate School for Integrative Sciences and Engineering, National University of Singapore, Singapore, 119228 Singapore

**Keywords:** C1D, Double-strand breaks, Homologous recombination, Condensin, Exosome, Apoptosis

## Abstract

Research on the involvement of C1D and its yeast homologues Rrp47 (*S. cerevisiae*) and Cti1 (*S. pombe*) in DNA damage repair and RNA processing has remained mutually exclusive, with most studies predominantly concentrating on Rrp47. This review will look to reconcile the functions of these proteins in their involvement with the RNA exosome, in the regulation of chromatin architecture, and in the repair of DNA double-strand breaks, focusing on non-homologous end joining and homologous recombination. We propose that C1D is situated in a central position to maintain genomic stability at highly transcribed gene loci by coordinating these processes through the timely recruitment of relevant regulatory factors. In the event that the damage is beyond repair, C1D induces apoptosis in a p53-dependent manner.

## Background

Eukaryotic cells harbor intricate and highly complex regulatory systems for survival. One of the primary causes of cell death is DNA damage, which ensues from spontaneous defects in these regulatory processes or as a result of other external insults, such as UV irradiation or toxins. DNA damage is usually resolved through the activation of DNA damage response (DDR) pathways that safeguard the genome and prevent genomic instability. The DDR is highly complex, and increasing evidence points to extensive overlap and feedback loops among the various proteins involved in promoting apoptosis, growth, and other cellular signaling networks [1]. Genes that are highly transcribed are particularly prone to genomic instability, and there is similarly a need to survey RNA quality at these highly transcribed regions, particularly at sites with repetitive sequences. This is achieved by RNA processing, and recent findings have demonstrated significant crosstalk between RNA processing and DDR activity in response to genotoxic stresses, as well as in the modulation of chromatin compaction in the transcribed sequences.

The DNA double-strand break repair protein, C1D, and its yeast homologue, Rrp47, have roles in RNA processing as well as in DNA damage repair. C1D physically interacts with proteins that function in these pathways as well as in the induction of apoptosis should DNA repair be unattainable. Another C1D homologue, Cti1, however, has distinct interactions with factors modulating chromatin condensation. Here, we will explore the data to suggest a central and overlapping role for C1D in the maintenance of these discrete processes.

## The DNA damage response (DDR)

Many hereditary diseases and spontaneous cancers arise as a result of alterations to genomic integrity (DNA mutations, chromosomal rearrangements, and aneuploidy). These genomic disturbances tend to be caused by defects in the replication process, recombination events during meiosis, or as a result of defects that arise during RNA processing. Damage can also be from exogenous sources such as cytotoxic agents that cause DNA double-stranded breaks (DSBs), or the introduction of DNA interstrand crosslinks (ICLs), UV and ionizing radiation (IR) as well as microbial and viral toxins. DSBs are highly toxic lesions and one of the most severe forms of DNA damage.

DNA damage is normally resolved through activation of DDR pathways, which recruit specific repair factors, halt the cell cycle, and invoke changes in the timing and expression of genes involved in DNA repair, cell-cycle control, and apoptosis [2]. In mammalian cells, DSBs are primarily repaired through one of two pathways: (1) homologous recombination (HR), which guides the repair of broken strands using a homologous stretch of DNA, or (2) non-homologous end-joining (NHEJ), which mediates the direct re-ligation of broken DNA ends. NHEJ is usually employed when the machinery required for HR is absent; however, this type of repair mechanism risks losing the proper sequence structure and can introduce errors, and thus is not the primary repair mode used by the cell (for specific pathway details, see [2]). HR is active mainly during the S- and G2 phases, whereas NHEJ does not appear to be restricted to a particular phase of the cell cycle [3]. HR is initiated by the MRN complex, composed of Mre11, RAD50 and NBS1, which can directly recognize breaks in the DNA to initiate HR-mediated repair [4]. The MRN complex orchestrates strand resection and recruits other RAD proteins (RAD51, RAD52) to search for the homologous section and mediate strand invasion [5]. Checkpoint proteins are also activated in response to DNA damage to arrest the cell cycle and allow time for DNA repair [6]. Ataxia telangiectasia, mutated (ATM), along with two other checkpoint kinases, ATR (ATM and Rad3-related), and DNA-dependent protein kinase (DNA-PK), are activated to regulate specific aspects of the DDR [2], with considerable convergence noted among their signaling cascades [6].

The DDR has a direct impact on genomic stability. Under normal conditions, efficient sensor systems help the cells to detect any potential DNA insult and elicit the proper cellular response. But a single DSB, if not properly repaired, can cause chromosomal aberrations such as translocations and deletions, which, in turn, result in a loss of heterozygosity, genomic instability, and, oftentimes, malignant transformation [3]. Indeed, the targeted disruption of factors involved in DDR is often utilized in synthetic lethality approaches to kill cancer cells via the concomitant destabilization of synergistically acting DNA repair pathways. For example, in cells that are deficient in HR repair mechanisms, such as following BRCA1 loss, the further disruption of polymerase θ, which is essential for microhomology-mediated end-joining, leads to cumulative death of the cancer cells [7, 8].

## The nucleolus and the exosome

The nucleolus, is a major sub-compartment within the nucleus that is important in the control of cell cycle and proliferation, stress sensing, tumor surveillance, DNA damage repair, protein stability and sequestration, and apoptosis [9]. The nucleolus houses the machinery required for the synthesis of ribosomal RNA (rRNA) and for the assembly of ribosomes. However, it is also involved in the assembly of protein-RNA complexes known as signal recognition particles (SRPs), as well as in the modification of transfer RNAs and in sensing cellular stress [10]. In the event of DNA damage or following nucleolar stress, several nucleolar proteins will relocalize to the nucleoplasm to support DDR pathway activities, and this response has led to the hypothesis that the nucleolus acts as a storage site for these proteins. Indeed, nucleolar sequestration—the capture and localization of proteins within the nucleolus through the stable interaction with anchored proteins, such as NPM1 or nucleolin—prohibits proteins from interacting with their targets, and thus acts as a key regulatory mechanism inside the cell [9, 11–13]. Interestingly, whereas nucleolar proteins migrate to the nucleoplasm during stress, few proteins migrate in the reverse direction [14, 15], and this lack of bidirectional flux is a proposed to be a form of surveillance that ensures the correct synthesis and assembly of ribosomal units. Indeed, in cases where there is a misassembly or improper synthesis, ribosomal synthesis is inhibited and there is a reorganization of nucleolar components to slow or arrest the cell cycle until a sufficient number of functional ribosomes are synthesized, or to allow time for the cell to trigger apoptosis pathways [9, 16, 17].

The RNA exosome (or PM/Scl complex) is a multiprotein complex found within the nucleolus of eukaryotic cells and archaea, and is responsible for the maturation of rRNAs, small nucleolar RNAs (snoRNAs), and small nuclear RNAs (snRNA) by limited trimming of the extended 3′-end of their nascent transcripts. Pre-rRNA undergoes processing into rRNA within the nucleolus with the aid of non-ribosomal proteins and snoRNAs within the exosome [10]. The exosome is also involved in the destruction and removal of RNA fragments and cryptic unstable transcripts (CUTs) [18], and is involved in RNA turnover and surveillance pathways, including the turnover of precursor transfer, messenger and ribosomal RNAs (pre-tRNAs, pre-mRNAs, pre-rRNAs) and the elimination of misprocessed RNAs [19].

The exosome assembles into a ring-like barrel composed of nine subunits. Despite its clear role in RNA processing and degradation, the exosome is almost completely devoid of function in the absence of its various cofactors [20]. Within this multiprotein complex, two proteins endow the exosome with its catalytic activity, the 3′-5′ exonuclease, Rrp6, which is confined to the nucleus, and the RNase Rrp44, also known as Dis3, which is the only active RNase in the complex [20, 21]. Rrp6 (PM/Scl-100 in eukaryotes) was the founding member of the exosome [22, 23] and is just one of several accessory proteins that is stably associated with this structure [24]. Rrp6 interacts with its obligatory binding partner, Rrp47 (C1D in eukaryotes), another important, stably associated, accessory protein. For years, it has remained unclear how the exosome and its associated factors can recognize and process so many different types of RNA, and it has only been in recent times that studies have been able to point to the role that these accessory co-factors play in not only recruiting other cofactors to the active site [25, 26] but also in facilitating exosome substrate recognition [27]. These key proteins and how they regulate RNA processing events will be discussed in the next sections in more detail.

Increasing evidence points to intimate links between RNA processing and the activation of DDR pathways [28]. This cellular rewiring is largely mediated by post-transcriptional mechanisms that regulate mRNA processing and metabolism [29]. For example, proper activation of the DDR pathway is ensured by the actions of the pre-mRNA splicing factors that prevent exon skipping and safeguard against DNA damage. Other DNA repair proteins, in turn, will provide regulatory feedback signals to control this splicing process [30]. These non-canonical functions of splicing factors and DNA repair proteins ensure genomic stability, and provide just some of the evidence that links these two major cellular functions. Intriguingly, proteins like C1D have been shown to be involved in RNA processing and DDR, and is thus appear to be appropriately situated at the nexus to coordinate these two processes.

## The C1D family of proteins

The C1D family of proteins comprises C1D homologues as well as several other proteins that each contain one or two Sas10/C1D domains, including C1D, Rrp47 and Cti1, as well as Sas10, Lcp5 and neuroguidin [31]. C1D, the prototypical member, is a small, 16-kDa mammalian nuclear matrix protein involved in higher order chromatin folding and tight DNA binding [32]. C1D was first identified as a corepressor of the nuclear hormone receptor repression complex [33] and has since been shown to be ubiquitously expressed in human tissues [34]. Its budding yeast (*Saccharomyces cerevisiae*) homologue, Rrp47, also known as Lrp1 or yC1D, is a 21-kDa protein that was identified through immunoaffinity purification of exosome complexes [35]. Both C1D and Rrp47 have been shown to bind DNA and RNA [24, 36, 37] and are involved in DNA repair mechanisms, RNA processing events, and aspects of translational control. They also both lack any characterized domains, aside from the Sas10/C1D domain, and it has been suggested that this domain may act as a docking site for proteins to interact with RNA and DNA concurrently [31].

Sas10 and Lcp5 are U3 small nucleolar ribonucleoproteins (snoRNPs) involved in 18S rRNA synthesis [38, 39], that, along with about 26 other proteins, constitute the multisubunit small subunit (SSU) processome. SnoRNPs are essential for yeast viability and are required for rRNA methylation and folding as well as ribosome function [40]. Mutations in LCP5 have been shown to be responsible for defects in pre-rRNA processing [38] and Sas10p depletion causes a severe reduction in 18S rRNA levels, without affecting 25S rRNA [39]. Neuroguidin, on the other hand, is a eukaryotic initiation factor 4E and CPEB binding protein [41], whereas Cti1, as will be discussed later, is a *Schizosaccharomyces pombe* homologue of C1D that binds to condensin and is essential for cell viability [42]. Interestingly, Sas10 is also reported to be closely connected with the structure of silenced chromatin in *S. cerevisiae* [43].

### Yeast Rrp47

Within the nucleolus of eukaryotes, RNA polymerase I transcribes a single precursor RNA that will be used to process 18S, 5.8S and 25S/28S rRNAs. Studies in yeast have shown that specific exosome components and associated proteins, such as Rrp47, are vital for such processing. Indeed, depletion of Rrp47 causes an accumulation of 3′-extended 5.8S rRNA as well as early pre-rRNA cleavage, which reduces 18S and 25S rRNA maturation [24]. Yeast Δ*Rrp47* mutants also display defects in DNA repair [44, 45].

Yeast Rrp47 is one of two nuclear RNA-binding proteins that interacts with the nuclear exosome; the other is M-phase phosphoprotein 6 (Mpp6) [18]. As mentioned above, Rrp47 also interacts with the exosome subunit Rrp6, an RNase D-related 3′ to 5′ exoribonuclease [46], with the two proteins binding directly through their Sas10/C1D and N-terminal PMC2NT domains, respectively [37, 47]. Rrp6 is found only in the exosome and, along with Rrp44 (also known as Dis3), provides this otherwise catalytically inert exosome with ribonuclease activity [48]. The Rrp47–Rrp6 complex specifically promotes Rrp6-mediated processes, such as nuclear RNA quality control, CUT degradation, and 3′-end maturation of stable RNAs [35, 49], and Rrp47 requires Rrp6 to localize to specific mRNA targets. Rrp47 is also reported to facilitate the maintenance of Rrp6 protein levels and the complex formed by these two proteins provides redundant exonuclease activities for the 3′ end maturation of box C/D snoRNA [47]. Depletion of either Rrp47 or Rrp6 results in similar RNA processing defects [35, 50], and there is evidence to suggest that the stability of Rrp47 is dependent on the proper expression of Rrp6 [47, 51, 52]. However, analyses show that when uncoupled from Rrp6, Rrp47 function is reduced but not completely blocked, suggesting that Rrp47 is not reliant on Rrp6 and can still function in Rrp6-independent RNA processing [52]. Furthermore, it seems that Rrp47 and Mpp6 are functionally redundant in pathways that target RNA degradation [52].

The Sas10/C1D domain of Rrp47 is critical for normal yeast growth and, although it is sufficient for protein function in vivo, in vitro studies suggest that a C-terminal lysine-rich stretch is also required for RNA binding [53]. The combined N-terminal domains of Rrp6 and Rrp47 provide a grooved surface for the binding of the helicase Mtr4 [25, 26, 54], and crystallography of the Rrp6–Rrp47 complex suggests that it is tethered to the top of the exosome by its N-terminus [26]. Yeast Mtr4p is an RNA helicase and functions as an auxiliary protein in most exosome functions [54, 55]. Mtr4 is vital for yeast cell viability, and is recruited by adaptor proteins, Nop53 and Utp18, to specific pre-ribosomes [27]. Unlike the other cofactors, Mtr4 has numerous roles within the RNA exosome, and its arch domain acts as a docking site for many RNA exosome cofactors that are involved in dictating specificity [27]. It is possible that Nop53 and Utp18 are involved in delivering RNA to the exosome via Mtr4 and the Rrp6–Rrp47 complex.

In *S. cerevisiae*, Rrp6–Rrp47, Mtr4 and Mpp6 combine with the exosome-10 complex in vitro to form a 14-subunit complex [25]. It has been hypothesized that, during RNA degradation, RNA binds to the top of the Rrp6–Rrp47 dimer but is then passed onto Rrp44 in a so-called “handover”, for processing. This initial binding step, however, suggests a previously unidentified role for Rrp6 in RNA recognition. During later RNA processing stages, after the bulk of the RNA particle reaches the top of the exosome, Rrp6–Rrp47 and also likely Rrp44 are then thought to undergo conformational changes in order to avoid steric clashes and, together with Mtr4 and Mmp6, facilitate the extraction the final 30-nucleotide extension. These hypotheses implicate a “division of labor” amongst the RNAases [26] and lend some insight into how the exosome can process so many forms of RNA while itself bearing such little RNase function and so few components.

The structure of Rrp6 from *Trypanosoma brucei* was solved recently with its binding partner *T. brucei* Exosome Associated Protein 3 (*Tb*EAP3), a potential orthologue of Rrp47 that shares about 21 % identity. *Tb*EAP3 lacks the Sas10/C1D domain and the lysine-rich residues yet it still is capabale of heterodimer formation with TbRRP6 in vitro and its biochemical behavior suggests its role as a platform protein for the binding of nucleic acids, similar to that of C1D. It is possible that *Tb*EAP3 could be a new member of the C1D family of proteins [56].

In addition to its role in RNA processing, Rrp47 demonstrates strong binding to DNA [37] and has been shown to play a role in DNA repair [44, 45], particularly in the 3′-end processing step in NHEJ, which is important for the proper union of DNA ends. Indeed, Rrp47 mutants showed approximately 50 % inappropriate repair of 3′-overhanging ends, suggesting that Rrp47 suppresses the utilization of error-prone DSB repair pathways in the cell. Furthermore, the Rrp47 mutants showed a two-fold reduction in HR repair; albeit the reduction was not as significant as that of the Rad52 mutant, which is an essential protein involved in HR pathways. Thus, overall, Rrp47 appears to maintain genome integrity by reducing the frequency of mutations being introduced during repair.

### Human C1D

In yeast, a set of conserved proteins is associated with the exosome and Rrp47. These same human orthologues have been identified for C1D: the catalytic exosome component PM/Scl-100 (Rrp6 in yeast), the nucleolus-specific exosome cofactor hMPP6 (Mpp6 in yeast) and hMtr4 (Dob1 or Mtr4 in yeast). Similar to its yeast homologue, C1D has a conserved role in RNA processing in association with the exosome. C1D binds efficiently to PM/Scl-100, which suggests that it is dependent on PM/Scl-100 to enter the nucleus and to accumulate in the nucleolus [24]. hMtr4p and the complex formed by PM/Scl-100, C1D and hMPP6 are required for 5.8S rRNA maturation [24, 57], with evidence to show that hMPP6 interacts with hMtr4 before binding to PM/Scl-100 [24].

C1D demonstrates high-affinity binding to DNA, even under denaturing conditions [58], and, like Rrp47, C1D has also been implicated in DNA binding and DNA repair pathways. C1D has been shown to interact with thyroid hormone receptor and RevErb orphan receptor as a co-repressor of DNA transcription [33]. C1D has been shown to interact with DNA-dependent protein kinase (DNA-PK) [59] in response to DNA damage [60], as well as in p53-dependent apoptosis [34, 61], as explored below.

#### C1D in DNA damage repair

DNA-PK is a serine/threonine kinase, with an N-terminal PI3 kinase motif and a leucine zipper (LZ) region in its C-terminal catalytic subunit (CS). The kinase has an important role in NHEJ as it localizes to DSBs and acts as a scaffold to organize a DNA repair complex through V(D)J recombination [62]. As mentioned earlier, DNA-PK has been reported to bind to C1D, with evidence suggesting that C1D is an efficient substrate for DNA-PK in vitro and in vivo [59]. The binding is thought to be direct, with C1D shown to bind to the LZ region of DNA-PK_CS_ [59]. However, this has been challenged by others, as DNA-PK harboring specific mutations in its LZ region is still able to bind to C1D and relocalize to the nuclear matrix [62]. Cells bearing a mutation in the LZ region, however, show considerably increased sensitivity to ionizing radiation, and appear to be less proficient at V(D)J recombination than their wild-type counterpart. Thus, it has been suggested that the LZ region contributes to DNA affinity rather than the catalytic function of the protein [62].

DNA-PK also comprises a DNA-binding Ku heterodimer that binds to DSBs and initiates NHEJ [63, 64]. Ku serves as a scaffold for the recruitment of canonical factors involved in NHEJ, such as DNA-PKcs [64] and, itself, has a high affinity for double-stranded DNA (dsDNA) ends [65]. Indeed, the DNA–Ku complex is reported to interact with dsDNA and facilitate DNA-PKcs kinase activity [66, 67]. Ku binding is, however, unaffected by mutations in the C1D-binding LZ motif of DNA-PKcs. Hence, C1D is thought to serve as an activator of DNA-PK in a manner that is independent on the activity of Ku [59]. Ku, and possibly C1D, targets DNA-PKcs to sites of DNA damage through direct binding with DNA to alter the availability of DNA ends [62].

C1D has also been implicated in DNA damage repair following UV radiation, with C1D expression dependent on xeroderma pigmentosum B (XPB) [60], an important component of the nucleotide excision repair (NER) pathway. A direct interaction between C1D and XPB is required to facilitate DNA repair, and this further implicates C1D in DNA repair mechanisms. However, C1D is not sufficient to fully rescue UV-induced damage, suggesting that other factors are at play. C1D has also been shown to interact stably with TRAX (translin-associated factor X), also in response to UV irradiation, and compete with its binding to Translin at sites of DSBs [68]. Translin is a DNA/RNA binding protein that specifically recognizes a consensus DNA sequence at breakpoint junctions of some types of chromosomal translocations, particularly those associated with lymphoid cancers [69]. It is believed that C1D has a protective effect in preventing the association of the TRAX/translin complex with DNA and thus inhibits recombination during times of DNA damage [68].

PM/Scl-100, the Rrp6 homologue, was also recently shown to directly regulate HR-dependent DSB repair via recruitment of the single-stranded DNA-binding HR protein RAD51. The depletion of Rrp6-family proteins from human and fly resulted in a hypersensitivity to irradiation, and this was associated with an impaired recruitment of RAD51 protein to sites of DNA damage, despite of not without affecting the level of the phosphorylation of histone H2AX (human) or H2Av (fly), the marker of DNA damage [70].

#### C1D in apoptosis

Physiological levels of C1D are essential for normal cell function (DSB repair and V(D)J recombination) but, unlike various other apoptosis-related genes, its overexpression has been linked with inducing apoptosis [34]. Indeed, C1D overexpression in tumor cells induces their apoptosis [34]. Inhibition of proteasome-dependent degradation pathways is able to enhance the expression of C1D in vitro and thereby increase apoptosis-induced cell death [61]. C1D, itself, is degraded by the ubiquitin-mediated processes [60] and, interestingly, cells expressing sub-threshold levels of C1D can overcome the ectopic overexpression of C1D by activating a proteasome-dependent mechanism for its degradation [61].

DNA-PK, in addition to its role in DSB repair, also phosphorylates several proteins involved in the apoptosis pathway, including p53 [71, 72]. C1D overexpression mimics the action of DSBs, leading to the activation of DNA-PK and p53, and resulting in apoptosis [34]. This is particularly interesting, as tumor cell survival seems to hinge on their inability to activate apoptosis pathways [71]. Controlling the transcription of C1D could therefore offer a significant step forward in gene therapy for apoptosis induction in tumor cells [71]. Upstream sequencing of C1D identified the presence of cis-acting repressing sequences (CRSs) that appear to be specific for repression of the C1D promoter. Rothbarth and colleagues further identified this upstream sequence as a LINE-1 element, and suggest that LINE-1 elements may function in the control of nearby genes [72]. Overall, inhibition of the proteasome-mediated degradation of C1D, together with the ability to control its transcription in tumor cells, may be concomitantly utilized for the treatment of tumor cells.

### Fission yeast Cti1

The *Schizosaccharomyces pombe* homologue of C1D was identified previously as an interacting partner in a two-hybrid screen with the SMC (structural maintenance of chromosomes) protein, Cut3 [42], and was consequently coined Cti1, which stands for condensin subunit CuT3 Interacting protein 1. Condensin is a conserved protein complex that is essential for chromosome condensation, functioning to compact the genome before cell division. The fission yeast complex comprises three non-SMC subunits Cnd1, 2 and 3, and two SMC subunits, Cut3 and Cut14 [73, 74] (a detailed review on the condensin complex can be found elsewhere [75]). Cti1 binds to condensin and is essential for cell viability, particularly during interphase growth [42]. It was previously shown that the ectopic expression of *cti1*^+^ could suppress the temperature sensitivity and hypersensitivity of a non-SMC mutant *cnd2*-*1* to DNA-damaging agents, including UV irradiation and the ribonucleotide reductase inhibitor, hydroxyurea [76]. This indicated that Cti1 functions, in part, to support the DDR function of the condensin complex. Interestingly, the localization of Cti1 is altered in response to DNA damage, re-localizing from the nucleolus to chromatin. Cti1 is one of few proteins that have been identified as a non-SMC accessory factor that interacts with the hinge motif of SMC proteins, which also includes Nse5 and Nse6, which bind to that of the hinge regions of Smc5/6 [77]. Although over a decade has passed, the mechanism by which condensin controls the condensation of the chromosome has yet to be fully elucidated.

## Connections between DDR and RNA processing: is there a broad overlapping role for C1D?

DDR and RNA processing are the two primary mechanisms operating throughout the genome, and only recent data has been able to show that the machineries that regulate these two processes are intimately related; albeit still poorly understood. In *S. cerevisiae*, mRNA surveillance machinery has been linked to DDR [45, 78]. Furthermore, senataxin, an RNA and DNA helicase, complexes with the exosome and targets it to sites of DNA damage induced by transcriptional errors [79].

Recent findings have linked RAD51, a factor involved in homologous recombination, with Rrp6 [70]. The group showed that the interaction is necessary for DDR foci formation, but also that the ribonucleic activity of Rrp6 was essential in this process. This not only confirms a role for Rrp6 in DDR but also links DDR with RNA processing functions in human and *Drosophila* [70]. Manfrini and colleagues (2015) also recently noted a role for Rrp6 and other RNA decay factors, Xrn6 and Trf4, in the repair of DSBs in budding yeast. ATR checkpoint kinase is essential in relaying the stress signal of DSB to facilitate HR-mediated DNA repair. ATR is activated upon the emergence of single-stranded DNA that are coated with replication protein A (RPA), and this occurs following the resection of DSBs. However, the loss of Rrp6 impairs the recruitment of RPA to single-stranded DNA and consequently, Mec1/ATR to DSBs, indicating that RPA regulates the localization and activation of the DNA damage checkpoint factors through recruitment of Mec1; albeit, the absence of this pathway did not affect the frequency of DNA resection at sites of DSBs [78]. In contrast to that observed in human and *Drosophila* [70], Manfrini and colleagues showed that inactivation of Rrp6 has no effect on Rad51/Rad52 association or on HR-mediated DSB repair in the budding yeast. Both papers, nevertheless, point to a link between DNA repair and RNA processing.

The improper processing of RNA that is transcribed from genes involved in DNA damage, such as ATM and ATR [80], causes a defect in DNA damage repair. Conversely, DNA damage can inhibit mRNA processing via the BRAC1/BARD1 complex, which physically interacts with cleavage stimulation factor (CstF), a protein that is necessary for the polyadenaylation of mRNA to ensure precise RNA quality control for effective translation [81–84]. Polyadenylation that is catalyzed by another complex, Trf4/Air2/Mtr4 polyadenylation (TRAMP), functions to regulate RNA quality control in conjunction with exosome components Rrp6 and Rrp47 [85].

RNA that results from gene transcription can hybridize with DNA to form aberrant R-loops, which, when not promptly resolved, lead to genomic instability. The accumulation of R-loops can also arise from an absence of RNA processing factors. These adducts are readily processed into DSBs by NER endonucleases, XPF and XPG, which are linked to the transcription-coupled repair (TCR) pathway [85]. This is consistent with the observation that DNA damage preferentially associates with highly transcribed regions [86]. In human tissues, R-loops can accumulate in the absence of BRCA2, suggesting a role for this repair protein in the processing of the R-loops [87]. Intriguingly, R-loops formed on enhancer sequence-derived non-coding RNA can be resolved by the exosome, as suggested by the enhanced rate of R-loop formation in cells depleted of exosome activity [88]. TCR is a canonical arm of NER [89]. However, a much wider range of transcriptionally linked pathways have been identified, and these mechanisms revealed that the relationship between co-transcriptional processes and DNA repair is much more intimate that previously realized.

Splicing is another important step in the processing of nascent RNA transcripts. Splicing factors have been found to regulate repair at highly transcribed genomic regions in cooperation with BRCA1 [90]. Besides interacting with the canonical DDR factors and preventing the formation of aberrant DNA/RNA hybrids, transcriptionally coupled complexes, particularly those involved in 3′ end processing (that also include the exosome), are also required for proper DDR; for example, cleavage factor 1 (CF1), which coordinates transcriptional termination and DNA replication [91]. Interestingly, some of the factors that act through TCR pathways perform RNA processing functions in the nucleoli and respond to DNA damage stress in the chromatin region, acting in a manner that resembles the function of Cti1/C1D. These factors include the base excision repair (BER) factor apurinic/apyrimidinic endonuclease 1 (APE1) [9] and nucleolin, which function to transcribe and process rRNA [92]. In the event of DNA damage, nucleolin is recruited by the MRN complex to sites of DSBs where it destabilizes nucleosomal compaction through its (recently identified) histone chaperone activity [93].

The recent work of Sutani et al. (2015) in fission yeast has shown that a persistence of transcription at coding genic regions during the mitotic phase of the cell cycle impedes precise chromosome segregation, probably due to the presence of unwound DNA, particularly at the 3′ end of the transcribed regions [94]. Consistently, the condensin complex was localized preferentially to the 3′ ends of genes and required for proper mitotic execution. The concomitant disruption of transcriptional machinery (for example, mediator component *med6*) sidestepped the requirement for condensin. Human condensin I complex was interestingly found to bind to active genes [94]. Since the C1D homologue Cti1 was initially isolated as a condensin-interacting factor [42], it is tempting to speculate a functional interaction between C1D and condensin. The inappropriate processing of the 3′ end of genes may act synergistically with improper compaction to undermine genetic integrity, and future experiments will be required to ascertain whether C1D has a role in these processes, in line with our previous knowledge of the role of Cti1.

## Conclusions

**Fig. 1:**
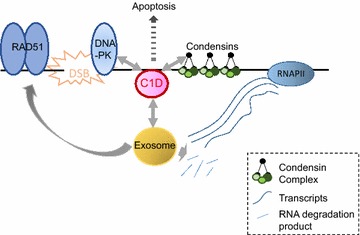
DNA double-strand repair protein C1D coordinates the exosome, condensin, and double-strand break (DSB) repair proteins in response to DSBs at highly transcribed genomic loci. Genomic instability at highly transcribed sites is common following RNA polymerase II transcription, and can be prevented by condensin-mediated stabilization of architectural integrity. RNA quality is also surveyed at these highly transcribed regions, particularly at sites hosting repetitive sequences. The formation of DSBs requires the prompt activation of repair pathways mediated by homologous recombination and non-homologous end joining machineries. When damage is unable to be repaired, the cells will induce apoptosis to prevent the cells from becoming carcinogenic. C1D protein physically interacts with proteins involved in all of these pathways, and we propose a coordinating role for the protein in maintaining genomic stability. Transcripts are indicated by *blue lines*; a defective transcript is indicated by a *crooked blue line*, which is being degraded by the exosome. *DNA-PK* DNA-dependent protein kinase, *RNAPII* RNA polymerase II

The ability of C1D to physically interact with proteins that function in DDR pathways and in RNA processing events as well as in the induction of apoptosis position it as an ideal candidate to coordinate processes that maintain genomic stability (Fig. 1). Indeed, C1D could coordinate the exosome, condensin, and DSB repair proteins in response to DSBs at highly transcribed genomic loci. Genomic loci that are nested within highly transcribed genes are especially prone to genomic instability. Such instability can be prevented by the preferential binding of condensin to stabilize the architectural integrity in the wake of RNA polymerase II transcription. The need for the surveillance of RNA quality is also proportionally increased at these highly transcribed regions, particularly at sites hosting repetitive sequences. The formation of RNA–DNA hybrids may result in a higher propensity for DSB formation, which will require prompt management by DSB repair machineries (HR and NHEJ) to preserve genomic stability. In the event that the damage is beyond repair, the cells must induce apoptosis to prevent carcinogenesis. Although much future work has yet to be undertaken, we propose a coordinating role for C1D in many of these aspects involved in maintaining genomic stability.

## Abbreviations

DSB: DNA double stranded break
HR: homologous recombination
NHEJ: non-homologous end-joining
MMEJ: microhomology-dependent end-joining
rRNA: ribosomal RNAs
snRNA: small nuclear RNAs
snoRNA: small nucleolar RNAs
pre-tRNAs: precursor transfer RNAs
pre-rRNAs: precursor messenger RNAs
CUT: cryptic unstable transcript
